# Huanglian Jiedu Decoction for treatment of multiple myeloma: A protocol for a systematic review and meta-analysis

**DOI:** 10.1097/MD.0000000000022378

**Published:** 2020-12-18

**Authors:** Na An, Yiwei Li, Xilian Huang, Can Chen, Yaping Xie

**Affiliations:** Department of Hematology, Affiliated Hangzhou First People's Hospital, Zhejiang University School of Medicine, Hangzhou, Zhejiang, PR China.

**Keywords:** Huanglian Jiedu Decoction, meta-analysis, multiple myeloma, protocol

## Abstract

**Background:**

Multiple myeloma can lead to lots of clinical problems including pain, fatigue, anemia, infections, renal failure, and so on. Huanglian Jiedu Decoction is a common conservative treatment for this disease in China. Therefore, we conducted a systematic review and meta-analysis to explore the efficacy of Huanglian Jiedu Decoction in the treatment of multiple myeloma.

**Methods:**

A systematic literature search for studies will be performed in 8 databases, including PubMed, Web of Science, Embase, the Cochrane library, ClinicalTrials.gov databases, Chinese National Knowledge Infrastructure Database, Wanfang database, and VIP database. The methodological quality of the included studies using the risk bias assessment tool of Cochrane. And the level of evidence for results is assessed by the Grading of Recommendations Assessment, Development, and Evaluation (GRADE) method. Statistical analysis is conducted with Revman 5.3.

**Results:**

This systematic review and meta-analysis will provide a synthesis of existed evidences for Huanglian Jiedu Decoction on multiple myeloma.

**Conclusion:**

The conclusion of this study will provide evidence to assess effectiveness of Huanglian Jiedu Decoction on multiple myeloma, which can further guide clinical decision-making.

**INPLASY registration number:**

INPLASY202060094

## 1 Introduction

Multiple myeloma (MM), which is characterized by uncontrolled growth of monoclonal plasma cells in the bone marrow, can lead to lots of clinical problems including pain, fatigue, anemia, infections, renal failure, and so on.^[1]^ Many symptoms of MM are vague and hard to pin down, and many patients seek clinical assistance just owing to a blood test. The International Myeloma Working Group by consensus defined the diagnostic criteria concerning MM in 2014. In brief, MM was defined as the presence of end-organ damage in parallel with the presence of an M-spike and/or monoclonal plasma cells.^[2]^ MM, which accounts for 1% of all cancers, is the most common hematological malignancy following lymphoma, with an estimated 242,802 to 30,330 new cases and 12,650 deaths in 2016.^[3–5]^ The median age of the patients at diagnosis is about 66 to 70 years old, of which 37% were younger than 65 years old. And MM is extremely rare among the people under 30 years old. The cause of MM is unknown, but it may be related to exposure with radiation, industrial/agricultural toxins.^[6]^ In recent years, the treatment measures, including chemotherapy, stem-cell transplantation, plasmapheresis, maintenance therapy, etc, have improved sharply, but there is no known cure for MM.

Due to some limitations of the mainstream treatments for MM, more and more attention has been paid to complementary alternative therapy. A host of monomers or compounds of traditional Chinese medicine have been used to treat MM in China. Additionally, quite many studies have proved that the use of traditional Chinese medicine can effectively improve the symptoms of patients suffering from MM.^[7–9]^ MM literally falls under the traditional Chinese medicine categories of “heat-toxin syndrome” in China. Huanglian Jiedu Decoction is a classical prescription described in Waitai Miyao, written by Wangtao during the Tang dynasty. It has always been a common compound used by traditional Chinese medicine doctors to treat “heat-toxin syndrome.” As shown in Table 1, Huanglian Jiedu Decoction is composed of rhizoma coptidis (Huanglian), radix scutellariae (Huangqin), cortex phellodendri (Huangbo), fructus gardeniae (Zhizi), all of which are reported to removing heat (Re) and detoxifying in China. Its main active components are berberine, baicalin, wogonoside, and gardenoside.^[10]^ Some new studies have shown that the mechanisms of action of Huanglian Jiedu Decoction in rats include antitumor activity,^[11]^ playing anti-inflammatory effects by reducing inflammatory factors such as IL-6, TNF-α, ect.^[12]^ Although previous studies estimated that Huanglian Jiedu Decoction are therapeutically effective for MM,^[13–15]^ These studies have the characteristics of small sample size and low methodology quality. Moreover, the clinical guidelines about MM also do not give recommendations on the treatment of Huanglian Jiedu decoction, even complementary alternative therapy.^[16–18]^ Hence, high-level quality evidence about Huanglian Jiedu Decoction intervention is urgently needed to help clinical decision-making for clinicians. After careful search, there are no published meta-analyses investing if the use of Huanglian Jiedu Decoction actually improves clinical outcomes. So this systematic review and meta-analysis was to evaluate the existing literature about Huanglian Jiedu Decoction treating for MM to determine the strength of evidence.

**Table 1:** Composition and function of Huanglian Jiedu Decoction.

Components	Role of Huanglian Jiedu Decoction	Function
Rhizoma coptidis (Huanglian)	Monarch (Jun)	Reduce the fire from upper-jiao
Radix scutellariae (Huangqin)	Minister (Chen)	Reduce the fire from middle-jiao
Cortex phellodendri (Huangbo)	Minister (Chen)	Reduce the fire from under-jiao
Fructus gardeniae (Zhizi)	Assistant (Zuo)	Clear heat and detoxify

## 2 Methods

This is a literature-based study, and thus no ethical approval and patient consent are required. The protocol of this study has been registered on the

International Platform of Registered Systematic Review and Meta-Analysis Protocol (INPLASY) (registration no. INPLASY202060094). In addition, it will be conducted according to the Preferred Reporting Items for Systematic Reviews and Meta-Analyses Protocols (PRISMA-P) statement guidelines.^[19]^

### 2.1 Literature research

**Figure 1:**
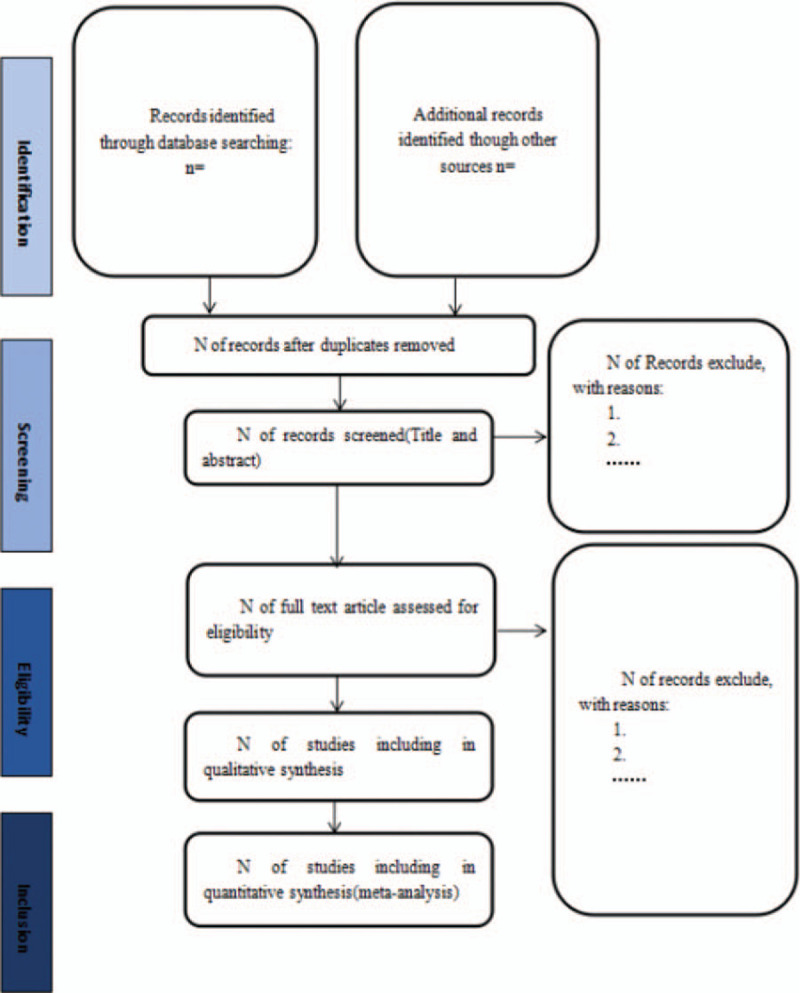
Flow diagram of literature search.

Relevant literature will be retrieved by electronically searching the following data sources: MEDLINE (by PubMed), Embase, Cochrane Library, ClinicalTrials.gov databases, Chinese National Knowledge Infrastructure Database, wanfang database, China Biology Medicine, and VIP database. There were no limits on study dates, language, publication type, or status. Search key terms were subjected to the following: “multiple myeloma,” “Huanglian Jiedu Decoction,” “Huanglian Jiedu Tang,” “Huanglian Jiedu Fang,” etc with the Boolean logic operator “AND,” “NOT,” and “OR.” Different search strategies will be used for the Chinese and foreign language databases. References cited in the relevant literature and other articles in the meta-analysis were also reviewed. Figure 1 shows the flow chart of this study selection and screening procedure.

### 2.2 Inclusion criteria

The retrieved literature is screened by 2 independent reviewers to evaluate eligibility using prespecified criteria, and any discrepancies are settled by discussion and consensus. First, the titles and abstracts of searched studies are screened. If these studies fail to meet the criteria, they will be excluded. Then, for studies that may or not be sure of the criteria, full texts are reviewed to examine whether each study meets the following criteria: randomized controlled trial; type of participants must be patients with symptomatic diagnosed MM; Huanglian Jiedu Decoction (including Huanglian Jiedu Decoction only and other treatments with Huanglian Jiedu Decoction) must be used for intervention. Control group is not restricted, but Huanglian Jiedu Decoction is not included. The primary outcomes include the following: progression-free survival, overall response rate, adverse event. When multiple time points were reported either in one particular report of a study or over the course of several articles from the same study, the longest follow-up period on treatment is considered in our study.

### 2.3 Exclusion criteria

The studies are excluded with the following characteristics: studies do not conform to the above criteria; both the treatment group and the control group included Huanglian Jiedu Decoction; studies are in the form of letters, abstracts, reviews, or comments; studies are impossible to extract relevant data.

### 2.4 Data extraction

The following information is independently extracted by 2 authors via a predesigned form: the name of first author, year of publication, country, sample size, patient characteristics in different groups (eg, age, gender of patients), disease course, description of the treatment and control interventions (eg, dose, length, setting), follow-up duration, outcome, as shown in Table 2. If a study just reports median, standard errors, 95% confidence interval (CI), or *P* values without standard deviations (SDs), we will transform these values into means or SDs through some statistical formulas. We will contact the authors by email or other ways if the data are missing, wrong, or unclear. Any disagreements between authors will be settled down by discussion.

**Table 2:** Basic characteristics of the included trials.

				Intervention				
Study ID	Sample size T/C (M/F)	Age (yrs) T/C	Course of disease E/C	Treatment group	Control group	Time to intervention	Follow-up	Outcomes
Fisrt author Publish time country	n (a/n-a)/n (2b/n-b)	c/d	e/f	Huanglian Jiedu Decoction or Huanglian Jiedu Decoction+other measures	Placebo or other measures	g	h	i, j, k

### 2.5 Quality assessment

**Figure 2:**
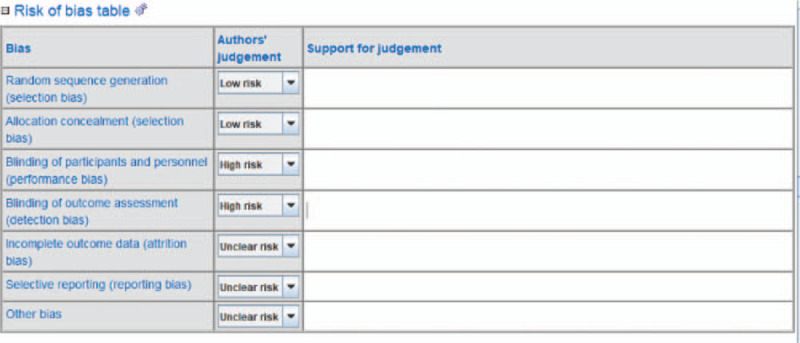
The risk of bias table.

We assessed the risk of bias of RCTs in this review using the Cochrane Collaboration Risk of Bias Tool provided by RevMan 5.3 software (Fig. 2).^[20]^ And risk of bias is assessed according to the Cochrane Handbook. For included study, types of bias are divided into 3 levels: low, unclear, high. Two authors independently assess the risk of bias of the included studies. The authors resolve any disagreements by discussion, including input from a third independent review author if required.

### 2.6 Data synthesis and statistical analysis

The dichotomous data is expressed as the relative risk. And the mean difference or standardized mean difference will be used to assess the difference in the continuous outcomes between the groups. Statistical heterogeneity across the included studies will be examined using the I^2^ statistic. Then, the authors will determine if there is a possibility of performing a meta-analysis. If I^2^ > 85%, the quantitative analysis will only be used. And with an 85% > I^2^ > 50% regarded as being indicative of the possibility of statistical heterogeneity, resulting in the selection of a random-effects model for merging of results. Otherwise, the fixed-effects model will be selected. To evaluate the robust of the results, the sensitivity analysis will also be conducted through excluding studies one by one. Furthermore, subgroup analyses will be performed if necessary. Forest plots and Egger regression test will be used to assess potential publication bias. Data regarding outcomes in the eligible trials are combined using the RevMan 5.3 and Stata 12.0, and the significance threshold will be a 2-sided *P* < .05.

### 2.7 Quality of evidence

The strength of the body of evidence will be determined according to the Grading of Recommendations Assessment, Development, and Evaluation method, which rate it into 4 levels: high, moderate, low, and very low. All operations are on this page: https://gradepro.org/

## 3 Discussion

Rates of Western medicine methods for MM have grown rapidly; however, more and more attention has been paid to complementary alternative therapies due to the attendant costs and complication risks. Huanglian Jiedu Decoction is one of the important intervention methods for these diseases in China.^[13–15]^ Besides, some reports about mechanism of Huanglian Jiedu Decoction have revealed its multichannel, which includes JAK2/STAT3, TLR4/MyD88 signaling pathway, etc, anti-inflammatory, and anti-cancer effect.^[11,21–23]^ After careful literature search, we found there is no quantitative meta-analysis on the treatment of MM by Huanglian Jiedu Decoction so far. It is, therefore, necessary to carry out a study to assess the efficacy, and help to propose the clinical recommendation for MM.

## Author contributions

**Conceptualization:** Can Chen.

**Data curation:** Na An.

**Formal analysis:** Xilian Huang.

**Investigation:** Yaping Xie.

**Methodology:** Yiwei Li, Xilian Huang.

**Resources:** Yaping Xie.

**Software:** Yiwei Li.

**Supervision:** Can Chen.

**Validation:** Yaping Xie.

**Visualization:** Xilian Huang.

**Writing – original draft:** Na An, Yiwei Li.

**Writing – review & editing:** Can Chen.
